# Birth prevalence of neural tube defects and associated risk factors in Africa: a systematic review and meta-analysis

**DOI:** 10.1186/s12887-021-02653-9

**Published:** 2021-04-21

**Authors:** Mohammed Oumer, Ashenafi Tazebew, Mezgebu Silamsaw

**Affiliations:** 1 Department of Human Anatomy, College of Medicine and Health Sciences, School of Medicine, University of Gondar, Gondar, Amhara Ethiopia; 2 Department of Epidemiology, College of Medicine and Health Sciences, Institute of Public Health, University of Gondar, Gondar, Amhara Ethiopia; 3 Departments of Pediatrics and Child Health, College of Medicine and Health Sciences, School of Medicine, University of Gondar, Gondar, Amhara Ethiopia; 4 Department of Internal Medicine, College of Medicine and Health Sciences, School of Medicine, University of Gondar, Gondar, Amhara Ethiopia

**Keywords:** Africa, Neural tube defects, Systematic review and meta-analysis

## Abstract

**Background:**

Neural tube defects are common congenital anomalies that result from early malformation in the development of the spinal cord and brain. It is related to substantial mortality, morbidity, disability, and psychological and economic costs. The aim of this review is to determine the pooled birth prevalence of neural tube defects and associated risk factors in Africa.

**Methods:**

The first outcome of this review was the pooled birth prevalence of the neural tube defects and the second outcome was the pooled measure of association between neural tube defects and associated risk factors in Africa. We systematically searched PubMed, PubMed Central, Joanna Briggs Institute, Google Scopus, Cochrane Library, African Journals Online, Web of Science, Science Direct, Google Scholar, and Medline databases. The heterogeneity of studies was assessed using the Cochrane Q test statistic, I^2^ test statistic, and, visually, using Forest and Galbraith’s plots. A random-effect model was applied to get the pooled birth prevalence of neural tube defects. Subgroup, sensitivity, meta-regression, time-trend, and meta-cumulative analyses were undertaken. The fixed-effect model was used to analyze the association between neural tube defects and associated risk factors.

**Results:**

Forty-three studies with a total of 6086,384 participants were included in this systematic review and meta-analysis. The pooled birth prevalence of the neural tube defects was 21.42 (95% CI (Confidence Interval): 19.29, 23.56) per 10,000 births. A high pooled birth prevalence of neural tube defects was detected in Algeria 75 (95% CI: 64.98, 85.02), Ethiopia 61.43 (95% CI: 46.70, 76.16), Eritrea 39 (95% CI: 32.88, 45.12), and Nigeria 32.77 (95% CI: 21.94, 43.59) per 10,000 births. The prevalence of neural tube defects has increased over time. Taking folic acid during early pregnancy, consanguineous marriage, male sex, and substance abuse during pregnancy were assessed and none of them was significant.

**Conclusions:**

The pooled birth prevalence of neural tube defects in Africa was found to be high. The risk factors evaluated were not found significant.

**Supplementary Information:**

The online version contains supplementary material available at 10.1186/s12887-021-02653-9.

## Background

Neural tube defects are common congenital anomalies that result from early malformation in the development of the brain and spinal cord [1–8]. It is the main cause of fetal loss and disabilities in neonates and it is considered a significant public health problem [3, 9–17]. The defects occur around 28th day after conception due to the failure of neurulation or alterations in the morphogenesis or histogenesis of the nervous tissue [1–3, 8].

Because of its complicated embryologic history, abnormal development of the spinal cord and brain is common [1]. Anencephaly, encephalocele, and spina bifida are the main types of neural tube defects [18–23]. The defects are correlated with substantial mortality, morbidity, disability, and psychological and economic costs [24]. Patients with these defects mostly have problems related to neurogenic bladder, orthopedic complications, kidney involvement, and hydrocephalus [25]. Patients with neural tube defects face lifelong physical problems that need lifetime medical care that add a significant burden to the affected patients and their families [25–27]. The challenges of parents begin with high distress at the time of diagnosis with defects during pregnancy and face either the grief of a termination/stillbirth or financial and emotional challenges of caring for a child with defects [25]. The lifetime direct medical costs and indirect costs for affected patients, parents, families, and at the national level are found very significant [25]. Prevention ensures that this multi-factorial burden does not have to happen at all [25, 28], and more literature is needed to fill the gaps. Worldwide, neural tube defects are among the top five most serious birth defects [13]. There are more than 400,000 births born affected by neural tube defects each year, causing around 88,000 deaths [9, 13, 29]. More than 10 % of newborns’ mortality happened due to the malformation of the spinal cord and brain [9]. In Africa, the most common birth defects are neural tube defects. It affects approximately 1–3/1000 births annually [18, 30–32]. In addition to its burden, stigmatization towards neural tube defects by the community has been documented elsewhere in Africa [13, 33, 34], affecting the quality of life of caring families with social, economic, and emotional distress.

The factors causing neural tube defects are genetic, nutritional, environmental, or a combination of these [1, 12, 13, 18, 35]. Epidemiologic studies have revealed that folic acid supplements and/or a vitamin-B taken before conception and continued for at least 3 months during pregnancy reduce the occurrence of neural tube defects [1, 23, 29, 36, 37]. Folic acid/folate intake can be increased either through consumption of a folic acid-containing supplement or consumption of staple foods fortified with folic acid in addition to a diet high in natural food of folate [13, 21]. Importantly, the folic acid fortification was revealed to significantly decrease the prevalence of the defects in countries around the globe [1, 37]. The prevalence of folic acid supplementation in Africa varies widely and showed folate deficiencies. Nevertheless, it is still difficult to conclude on the extent of folate deficiencies in Africa due to the limited amount of data available [38]. In addition, neural tube defects are influenced by certain drugs (e.g., valproic acid, if given during 4th-week development as the neural folds are fusing), prenatal factors (e.g., maternal infection or thyroid disorder, Rh factor incompatibility, and some hereditary conditions), presence of chronic disease during pregnancy, and substance use during pregnancy [1, 2, 12, 14, 18, 25, 39].

The aim of this systematic review and meta-analysis is to determine the pooled birth prevalence of neural tube defects and to identify the pooled measure of association between the neural tube defects and associated risk factors in Africa.

## Methods

The preferred reporting items for systematic reviews and meta-analysis (PRISMA) statements were adapted to report the present review of meta-analysis [40] (Supplementary file 1). The international prospective register of a systematic review (PROSPERO) registered (CRD registration number is CRD42020169443) this review (https://www.crd.york.ac.uk/).

### Review outcomes

The first outcome of this review was the pooled birth prevalence of neural tube defects. The second outcome was the pooled measure of association between neural tube defects and associated risk factors in Africa. Birth prevalence of neural tube defects is defined as the number of neural tube defect cases of live births and/or stillbirths at birth from the total number of births (live births and/or stillbirths) during the study period.

### Study eligibility criteria

The inclusion criteria for this review were published and unpublished studies in any period (the study period was not restricted for inclusion), and study designs that report the birth prevalence (live births and/or stillbirths) and/or associated risk factors of neural tube defects in Africa. Case reports, anonymous reports, editorials, and conferences were excluded. The study was excluded if the total number of cases as well as the total number of births were under-reported for the prevalence objective.

### Searching strategies and information sources

PubMed, PubMed Central, Google Scopus, Medline, Cochrane Library, JBI Library, Web of Science, Science Direct, Popline, CINAHL, African Journals Online, UCSF, WHO, and Embase databases were systematically searched up to April 18, 2020, for relevant studies. Grey literature and other sources were retrieved using Google and advanced Google Scholar searches. Reference lists, bibliographies, of identified studies were navigated for additional studies. The corresponding authors were contacted for missing important data. The primary search was performed in an advanced PubMed database, using Medical Subject Heading [MeSH] terms, (Supplementary file 2). Besides, the search in other databases was performed using the mentioned core search terms interchangeably (neural tube defects, newborns/live births/stillbirths, and Africa).

### Study selection

After retrieving all studies from the databases, we exported citations to the bibliographic software, Endnote Version 7 Software, to remove the duplicate studies. Then, the reviewers screened studies based on the abstract and title for possible inclusion. Two reviewers (MO and AT) independently considered the criteria (pre-determined selection criteria) to select studies. The first two authors, independent of each other, selected all articles. Studies were deeply reviewed entirely in order to identify the final included article.

### Methodological quality

We used the Joanna Briggs Institute (JBI) quality appraisal scale to assess the risk of bias in each study [41]. Essentially, two reviewers (MO and MS) independently assessed the quality of each study. Disagreements raised between reviewers were solved based on discussions or by taking the average score of the two reviewers. The JBI quality appraisal scales were adapted for the cohort studies, cross-sectional studies, case-control studies, and for the studies reporting the prevalence data (Supplementary file 3). The study was considered low risk if the study scored five and above points in all quality assessment items.

### Data extraction

After including the eligible studies, three reviewers (AT, MS, and MO) extracted all essential data independently using a standardized, pre-specified, data abstraction format. The pre-specified format minimized the reviewers’ conflict of interest in the data extraction process but for any discrepancy of interests raised, the discussion was used to solve raised issues. If necessary, the main author of the study was communicated.

The data extraction format included first author, study country, publication year, sample size, study duration, study design, prevalence period, study setting, birth outcome, the birth prevalence of neural tube defects, and associated risk factors (adjusted odds ratio with a confidence interval of the variables were taken based on available literature). Prevalence reports of all studies in the different denominators have been converted into per 10, 000 births to maintain uniformity. Then, we have used per 10, 000 prevalence estimates for reporting the findings of this review. The assessed factors were folic acid supplementation during early pregnancy, consanguineous marriage, male newborn, and substance abuse during pregnancy.

#### Meta-analyses

The data analyses were conducted using STATA Version 14 Statistical Software. The data were extracted in Microsoft Excel and it was exported into STATA 14 Software for further analyses. For all studies, the median value, interquartile range, and the minimum and maximum values of neural tube defects were calculated.

The heterogeneity between the studies was assessed using visual and statistical techniques. Visually, the Galbraith plot and Forest plot were used to assessing the presence of heterogeneity. The Q test and I-Squared (I^2^) test statistics were considered to examine the variations. The heterogeneity was declared as low, moderate, or high when the I^2^ test statistic result became 25 %, 50 %, and 75 %, respectively [42]. This review displayed that there was significant heterogeneity among studies (*P*-value < 0.001). Thus, we adopted the random effect model to get the birth prevalence of neural tube defects [43]. However, the analysis demonstrated that there was a non-significant heterogeneity in estimating an association, and the fixed-effect model was adopted to analyze the association between neural tube defects and factors [44].

Given the variations in estimating the pooled birth prevalence, we conducted a subgroup analysis based on identified covariates to reduce the heterogeneity. Random effect meta-regression analyses were accounted for to determine the source of heterogeneity. We performed the sensitivity analyses to evaluate the influence of the study on the overall pooled estimates (the outputs of influence/sensitivity analyses were displayed graphically as well). We performed the time-trend analysis in order to visualize the random variations in the time sequence. A meta-cumulative analysis was done to display the pattern of effects and to show the significance of cumulative effect over the publication years.

The publication bias was checked using Egger’s regression test (and Begg’s test) statistics [45, 46] and we declared the presence of significant publication bias if a *P*-value became less than 0.05. Egger’s plot and the funnel plot were also considered. The trim and fill analyses were considered to mitigate the publication bias.

## Results

**Fig. 1:**
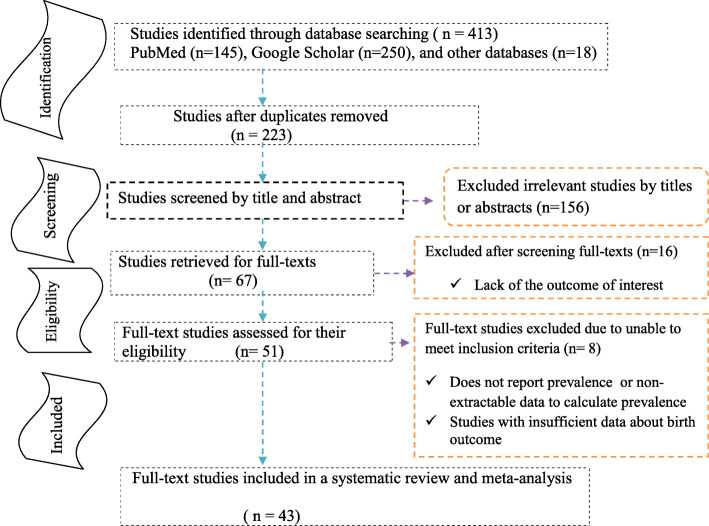
Study selection flow diagram, a figure adapted from the Preferred Reporting Items for Systematic Reviews and Meta-Analyses (PRISMA) group statement

The comprehensive search of databases yielded 413 studies about neural tube defects and associated risk factors in Africa. Of 223 unduplicated studies, we excluded 156 after reviewing the titles and abstracts. The remaining sixty-seven studies were screened and sixteen were excluded because of the outcome interests. Thus, fifty-one studies were assessed for eligibility, and 43 studies, were fulfilled the criteria, were included in this systematic review and meta-analysis (Fig. 1). All included original studies were cross-sectional (29), case-control (5), and prospective cohort (9) study designs [2–23, 47–68]. Of these studies, we used thirty-six for prevalence estimates and all these were cross-sectional and prospective study designs [2, 3, 5–17, 47–55, 57–68]. The total number of participants included was 6086, 384. Ten studies had been conducted in Ethiopia [2, 5, 9–11, 17–20, 64], five in Tunisia [3, 8, 21–23], eight in Nigeria [6, 12, 15, 16, 50, 51, 65, 66], two in Algeria [4, 7], six in South Africa [55, 59, 60, 63, 67, 68], two in Sudan [48, 62], and two in Ghana [49, 57]. Study was conducted in Kenya [13], Eritrea [14], Libya [52], Egypt [53], Cameron [54], Malawi [58], Tanzania [61], and Democratic Republic (DR) of Congo [47] (Table 1). The period prevalence, birth outcome, study setting, and study quality were presented in Table 2. In addition to studies explained in Table 2, studies done by Atlaw et al. [18], Berihu et al. [19], Aynalem et al. [20], Nasri et al. [21], Bourouba et al. [4], Kitova et al. [22], and Nasri et al. [23] were declared low risk.

**Table 1:** The characteristics of the studies included in the systematic review and meta-analysis, 2020

First author	Year	Country	Study design	Sample size	Duration/months	Prevalence per 10, 000 births
Gedefaw et al. [2]	2018	Ethiopia	Cross-sectional	8677	7	63
Nasri et al. [3]	2014	Tunisia	Cross-sectional	3,803,889	240	2
Adane et al. [5]	2018	Ethiopia	Cross-sectional	19,650	36	52
Anyanwu et al. [6]	2015	Nigeria	Cross-sectional	1456	9	27
Houchar et al. [7]	2008	Algeria	Cross-sectional	28,500	36	75
Berihu et al. [9]	2018	Ethiopia	Cross-sectional	14,903	9	131
Taye et al. [10]	2019	Ethiopia	Cross-sectional	76,201	6	40
Abebe et al. [11]	2020	Ethiopia	Cross-sectional	45,951	60	41
Nnadi et al. [12]	2016	Nigeria	Prospective	10,163	36	22
Githuku et al. [13]	2014	Kenya	Cross-sectional	6041	72	3
Estifanos etal [14].	2017	Eritrea	Cross-sectional	39,803	24	39
Toma et al. [15]	2018	Nigeria	Cross-sectional	1046	35	250
Audu et al. [16]	2004	Nigeria	Cross-sectional	2250	48	80
Legesse et al. [17]	2019	Ethiopia	Prospective	956	7	63
Nasri et al. [8]	2015	Tunisia	Cross-sectional	764,431	48	2
Atlaw et al. [18]	2019	Ethiopia	Case-control	462	6	–
Berihu et al. [19]	2019	Ethiopia	Case-control	617	9	–
Aynalem etal [20].	2018	Ethiopia	Case-control	180	7	–
Nasri et al. [21]	2015	Tunisia	Case-control	150	7	–
Bourouba et al. [4]	2018	Algeria	Case-control	130	12	–
Kitova et al. [22]	2013	Tunisia	Prospective	150	36	–
Nasri et al. [23]	2016	Tunisia	Prospective	132	9	–
Ahuka et al. [47]	2006	DR Congo	Cross-sectional	8824	96	10
Oumer et al. [48]	2016	Sudan	Cross-sectional	36,785	12	28
Alhassan et al. [49]	2017	Ghana	Cross-sectional	35,426	48	16
Alrede et al. [50]	1992	Nigeria	Prospective	5, 977	36	70
Ekanem et al. [51]	2008	Nigeria	Cross-sectional	127,929	276	5
Singh et al. [52]	2000	Libya	Prospective	15, 938	12	8
Mohammed etal [53].	2011	Egypt	Cross-sectional	5000	7	16
Njamnshi et al. [54]	2008	Cameron	Cross-sectional	52,710	120	19
Sayed et al. [55]	2008	South Africa	Prospective	53,000	9	10
Masamati et al. [58]	2000	Malawi	Cross-sectional	25,562	24	6
Venter et al. [59]	1995	South Africa	Prospective	10,380	40	36
Buccimazzaetal [60].	1994	South Africa	Cross-sectional	516,252	240	12
Kinasha et al. [61]	2003	Tanzania	Cross-sectional	34,000	24	30
Elsheikh et al. [62]	2009	Sudan	Prospective	18,378	12	35
Krzesinski etal [63].	2019	South Africa	Cross-sectional	93,609	72	7
Anyebuno et al. [57]	1993	Ghana	Cross-sectional	19,094	24	12
Adetiloye et al. [66]	1993	Nigeria	Cross-sectional	23, 438	120	5
Sorri et al. [64]	2015	Ethiopia	Cross-sectional	28, 961	36	54
Ugwo et al. [65]	2007	Nigeria	Cross-sectional	2, 891	48	128
Cornell et al. [67]	1983	South Africa	Cross-sectional	116, 859	60	9
Kromberg et al. [68]	1982	South Africa	Cross-sectional	29, 633	–	8

**Table 2:** The study period, setting, birth outcome, and quality of included studies in the systematic review and meta-analysis, 2020

First author	Birth outcome	Prevalence period	Study setting	Study quality
Gedefaw et al. [2]	LB + SB	2016	Institution-based	Low risk
Nasri et al. [3]	LB + SB	1991–2011	Institution-based	Low risk
Adane et al. [5]	LB + SB	2015–2017	Institution-based	Low risk
Anyanwu et al. [6]	LB	2013	Institution-based	Low risk
Houchar et al. [7]	LB + SB	2004–2006	Institution-based	Low risk
Berihu et al. [9]	LB + SB	2016–2017	Institution-based	Low risk
Taye et al. [10]	LB	2015	Institution-based	Low risk
Abebe et al. [11]	LB + SB	2011–2015	Institution-based	Low risk
Nnadi et al. [12]	LB + SB	2011–2013	Institution-based	Low risk
Githuku et al. [13]	LB	2005–2010	Institution-based	Low risk
Estifanos et al. [14]	LB + SB	2007–2011	Institution-based	Low risk
Toma et al. [15]	LB + SB	2013–2016	Institution-based	Low risk
Audu et al. [16]	LB	2000–2003	Institution-based	Low risk
Legesse et al. [17]	LB + SB	2018–2019	Institution-based	Low risk
Nasri et al. [8]	LB + SB	2008–2011	Institution-based	Low risk
Ahuka et al. [47]	LB	1993–2001	Institution-based	Low risk
Oumer et al. [48]	LB + SB	2014–2015	Institution-based	Low risk
Alhassan et al. [49]	LB + SB	2010–2014	Institution-based	Low risk
Alrede et al. [50]	LB + SB	1987–1990	Institution-based	Low risk
Ekanem et al. [51]	LB + SB	1980–2003	Institution-based	Low risk
Singh et al. [52]	LB + SB	1995–1996	Institution-based	Low risk
Mohammed et al. [53]	LB	2007	Institution-based	Low risk
Njamnshi et al. [54]	LB + SB	1997–2006	Institution-based	Low risk
Sayed et al. [55]	LB + SB	2004–2005	Institution-based	Low risk
Masamati et al. [58]	LB + SB	1998–1999	Institution-based	Low risk
Venter et al. [59]	LB	1989–1992	Institution-based	Low risk
Buccimazza etal [60].	LB + SB	1973–1992	Institution-based	Low risk
Kinasha et al. [61]	LB	2000–2002	Institution-based	Low risk
Elsheikh et al. [62]	LB + SB	2003–2004	Institution-based	Low risk
Krzesinski et al. [63]	LB + SB	2003–2013	Institution-based	Low risk
Anyebuno et al. [57]	LB + SB	1991–1992	Institution-based	High risk
Adetiloye et al. [66]	LB + SB	1982–1992	Institution-based	High risk
Sorri et al. [64]	LB + SB	2009–2012	Institution-based	–
Ugwo et al. [65]	LB + SB	2002–2005	Institution-based	–
Cornell et al. [67]	LB + SB	1975–1980	Institution-based	–
Kromberg et al. [68]	LB + SB	–	Institution-based	–

Key: *LB* Live births, *SB* Stillbirths

**Fig. 2:**
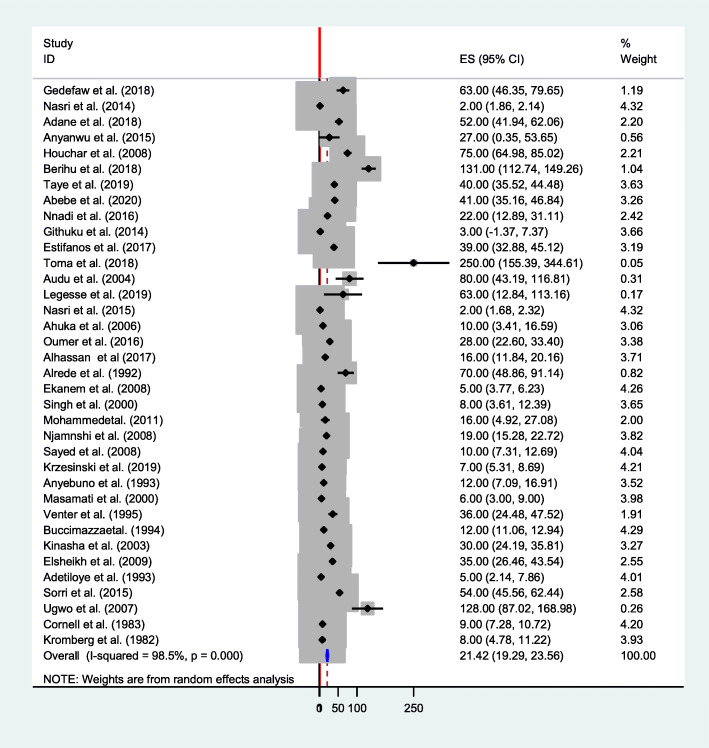
Forest plot showing the pooled prevalence of neural tube defects in Africa, 2020

The pooled birth prevalence of neural tube defects in the present meta-analysis was 21.42 (95% CI: 19.29, 23.56) per 10,000 births. A forest plot showed that there was significant heterogeneity across the studies (*P*-value < 0.001, I^2^ = 98.5%). Therefore, a random-effect model was applied to pool the overall prevalence [2, 3, 5–17, 47–55, 57–68] (Fig. 2). For thirty-six studies, the median value of neural tube defects was 24.5 and the inter-quartile range was between 8.5 and 53 per 10, 000 births. The minimum and maximum values were 2 and 250 per 10, 000 births.

Subgroup analyses based on the period prevalence, region/country, the birth outcome, and design was performed. The highest and the lowest prevalence rate was found in Algeria (75.0, 95% CI: 64.98, 85.02) and in Tunisia (2.0, 95% CI: 1.87, 2.13, per 10,000 births) (Supplementary file 4). Based on the birth outcome (I^2^ = 98.5%), the prevalence for live births only was 26.85 (95% CI: 13.43, 40.27) and for both live birth and stillbirths was 19.76 (95% CI: 17.49, 22.03) per 10,000 births. Concerning the study designs (I^2^ = 98.5%), the prevalence for the cross-sectional (21.01, 95% CI: 18.74, 23.27) was lower than the prospective cohort study designs (28.35, 95% CI: 17.53, 39.17, per 10, 000 births). Based on the period prevalence (I^2^ = 98.5%), the burden of neural tube defects for the period after 2010 was 49.55 (95% CI: 36.50, 62.61), 2001–2010 was 29.48 (95% CI: 22.10, 36.87), 1991–2011 was 2.0 (95% CI: 1.86, 2.14), 1991–2000 was 12.42 (95% CI: 6.46, 18.38), 1980–2003 was 5.0 (95% CI: 3.77, 6.22), and before 1990 was 10.65 (95% CI: 6.52, 14.77) per 10,000 births.

Sample size (*P*-value = 0.78), year of publication (*P*-value = 0.37), duration of the study in months (*P*-value = 0.74), study quality score (*P*-value = 0.69), study country (*P*-value = 0.03), study design (*P*-value = 0.84), birth outcome (*P*-value = 0.63), and period prevalence (*P*-value = 0.47) were analyzed for the source of heterogeneity and only study country was found statistically significant.

**Fig. 3:**
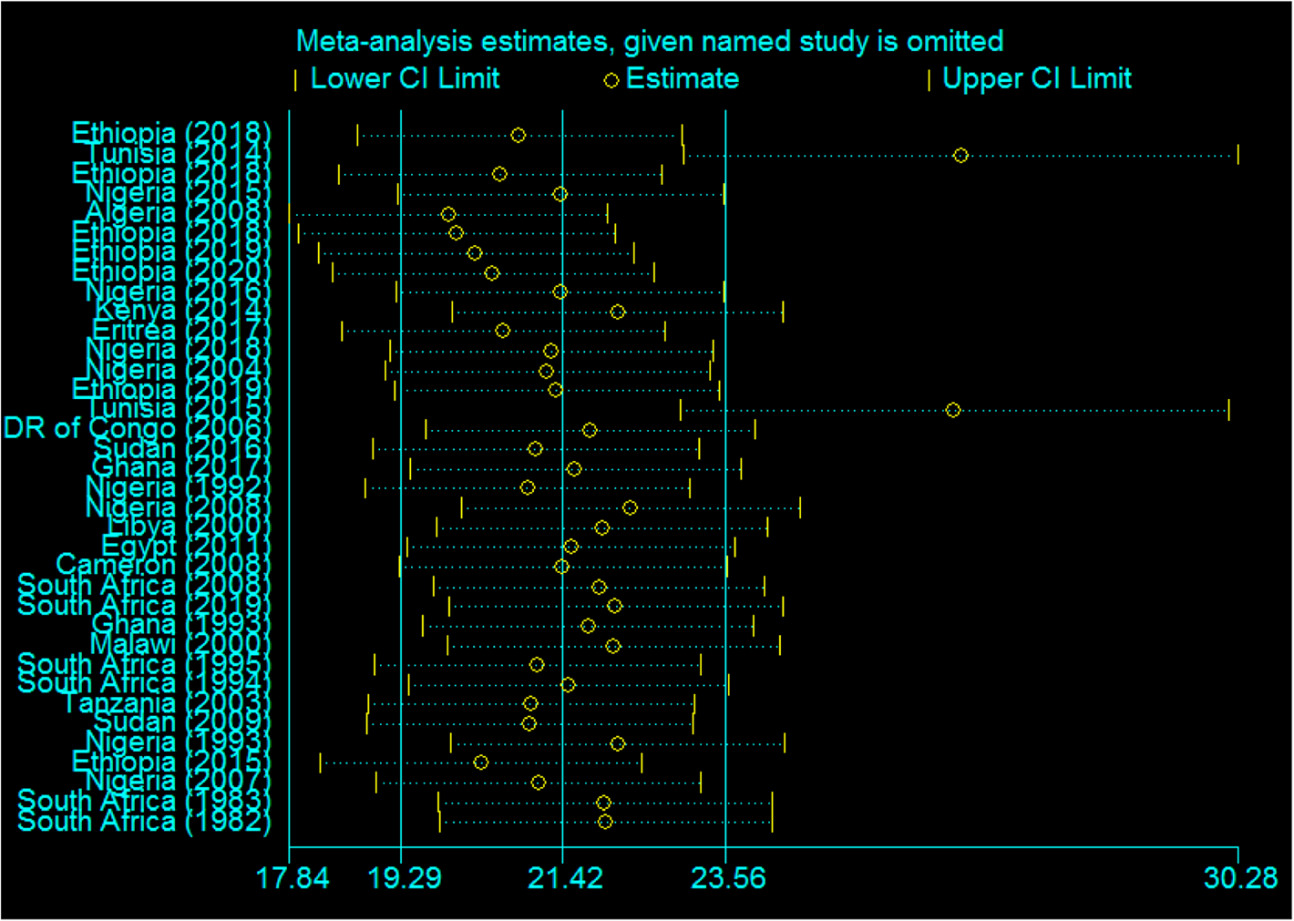
Sensitivity analysis showed the influence of each individual study in overall estimates in Africa, 2020

In the current systematic review and meta-analysis, except for two Tunisian studies (years 2014 and 2015), the influence of studies on the overall estimates was uniform (Fig. 3). Meta-influence estimates were analyzed by removing one article at a time and the uniform influence was displayed and the prevalence after removing only the 2014 Tunisia study was 26.64 (95% CI: 23.0, 30.28), and after removing only the 2015 Tunisia study was 26.56 (95% CI: 22.96, 30.16) (Fig. 3). If both studies are omitted together, the prevalence was 28.24 (95% CI: 24.22, 32.27) with uniform influence. Even if the whole analysis was repeated after omitting the two studies, the heterogeneity across studies was not decreased (97.5%, only 1% reduction). We looked at the effect of low-quality studies on the overall estimates by limiting those studies included in a meta-analysis. The meta-analysis estimate was found by including studies that only scored greater than or equal to five, high-quality studies; therefore, its pooled estimate was 22.31 per 10,000 births.

**Fig. 4:**
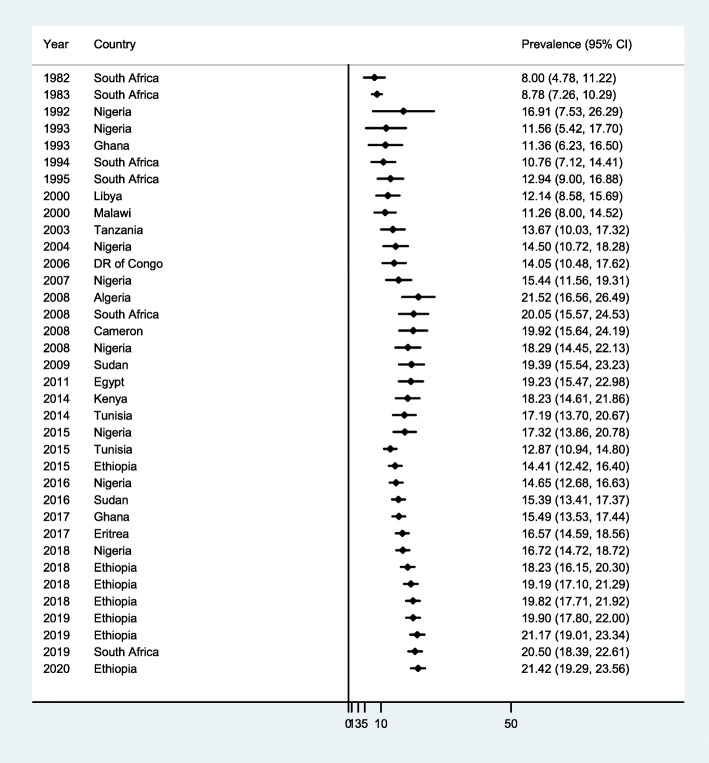
Meta-cumulative analysis showing cumulative effect of neural tube defects in relation to time in Africa, 2020

The relationship between the burden of neural tube defects and the study publication years from 1982 (8.0) to 2020 (41 per 10,000 births) was visualized using the time trend analyses. Besides, the pattern of effects on the time, from the year 1982 (8.0) to the year 2020 (21.42 per 10,000 births), was displayed using the meta-cumulative analyses and the cumulative effects of all studies were significant (Fig. 4).

**Fig. 5:**
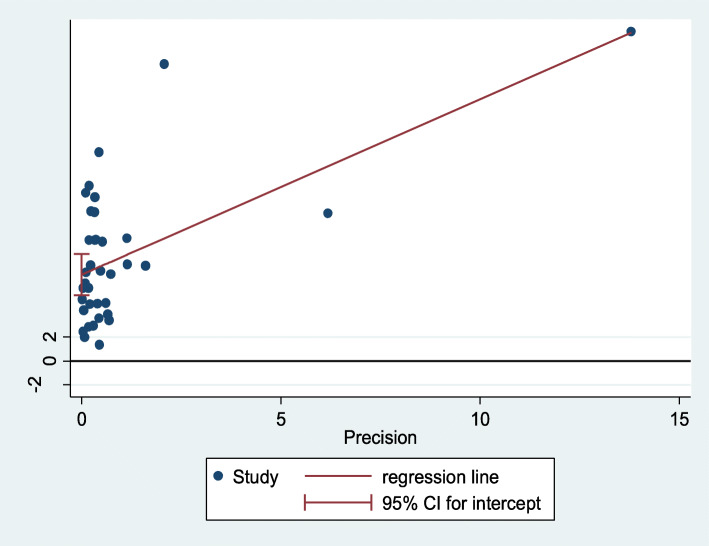
Egger’s publication bias plot, 2020

In estimating the birth prevalence, a significant publication bias was identified by Egger’s tests (*P*-value < 0.001) and its plot (Fig. 5). We conducted the trim and fill meta-analyses to adjust this bias. We analyzed fifty-five studies (19 articles were filled in the 36 studies) in the fill meta-analyses. As a result, the birth prevalence of neural tube defects using the random-effect model was 5.14 (95% CI: 2.90, 7.38) per 10,000 births. This adjusted estimate suggested a lower risk of bias than the original analysis. However, publication bias is still significant after fill and trim analyses have been done.

In this meta-analysis, folic acid supplementation during early pregnancy, consanguineous marriage, male newborn, and substance abuse during pregnancy (smoking, alcohol, especially) were the variables analyzed for association with neural tube defects. In estimating the association of all factors, there was no statistically significant publication bias among studies. Similarly, the Galbraith plot visualized that there was no heterogeneity among the studies. The summary of studies (odds ratio, confidence interval, etc.) included in the meta-analyses for an association was explained in Table 3.

**Table 3:** Summary of studies included in the meta-analysis for association with neural tube defects, 2020

First author	Year	Country	Associated factors	Odds ratio	95% confidence interval	
			Folic acid		UCI	LCI
Gedefaw et al. [2]	2018	Ethiopia		0.47	0.95	0.23
Bourouba et al. [4]	2018	Algeria		0.24	1.15	0.03
Anyanwu et al. [6]	2015	Nigeria		0.36	19.27	0.03
Atlaw et al. [18]	2019	Ethiopia		0.095	0.29	0.001
Berihu et al. [19]	2019	Ethiopia		0.48	1.04	0.2
Nasri et al. [8, 21]	2015	Tunisia		1.19	2.44	0.58
Nasri et al. [23]	2016	Tunisia		0.15	0.44	0.04
**Pooled/net odds ratio**				**0.51**	**2.29**	**0.11**
**Consanguineous marriage**						
Atlaw et al. [18]	2019	Ethiopia		5.54	20.9	1.47
Nasri et al. [8, 21]	2015	Tunisia		2.09	6.1	0.76
Kitova et al. [22]	2013	Tunisia		2.46	6.37	0.95
Nasri et al. [23]	2016	Tunisia		2.59	11.9	0.69
Nasri et al. [8, 21]	2015	Tunisia		1.27	4.59	0.35
**Pooled/net odds ratio**				**2.41**	**18.47**	**0.31**
**Male newborn**						
Gedefaw et al. [2]	2018	Ethiopia		0.56	0.94	0.33
Nasri et al. [3]	2014	Tunisia		0.68	0.79	0.59
Anyanwu et al. [6]	2015	Nigeria		0.92	12.77	0.07
Houchar et al. [7]	2008	Algeria		0.7	0.92	0.52
Atlaw et al. [18]	2019	Ethiopia		0.72	1.37	0.38
Aynalem et al. [20]	2018	Ethiopia		0.58	1.14	0.3
Nasri et al. [8, 21]	2015	Tunisia		0.49	1.27	0.19
**Pooled/net odds ratio**				**0.67**	**1.06**	**0.42**
**Substance abuse during pregnancy**						
Atlaw et al. [18]	2019	Ethiopia		11.08	62.7	1.96
Berihu et al. [19]	2019	Ethiopia		10.3	88.5	1.19
Aynalem et al. [20]	2018	Ethiopia		0.56	1.5	0.21
**Pooled/net odds ratio**				**1.52**	**33.28**	**0.07**

Key: The different numbers of articles in different analysis/variables is due to a lack of similarity in studies reporting the risk factors

Taking folic acid during early pregnancy (Pooled OR (Odds Ratio) = 0.51, 95% CI: 0.11, 2.29), consanguineous marriage (Pooled OR = 2.41, 95% CI: 0.31, 18.47), male sex (Pooled OR = 0.67, 95% CI: 0.42, 1.06), and substance abuse during pregnancy (Pooled OR = 1.52, 95% CI: 0.07, 33.28) were assessed and none of them was statistically significant (Supplementary file 4).

## Discussion

The present systematic review and meta-analysis were conducted to assess the pooled birth prevalence of neural tube defects and to identify the risk factors associated with the occurrence of neural tube defects. This review revealed the pooled birth prevalence in Africa and it evaluated the risk factors (folic acid uptake, consanguineous marriage, male newborn, and substance abuse during pregnancy) for association with neural tube defects. The hidden burden of neural tube defects is very high in Africa. The primary data research and systematic review/meta-analysis that show this burden are scarce. However, the effects of the defects are related to substantial mortality, disability, and psychological costs and it is an important public health problem [24, 69–73].

The pooled birth prevalence of the neural tube defects in the present meta-analysis was found 21.42 per 10,000 births with a range of 19.29–23.56. Different prevalence rates have been reported by the review conducted in Indian [74], Latin America [75], and worldwide [24]. Variation in estimates was also observed in reviews reported elsewhere [69, 70, 74, 75]. The prevalence of the defect remains high in less-developed countries of Africa, Latin America, Asia, and the Far East [1, 71–73]. The variation in estimates may be due to the difference in countries’ health policy, income levels, and the institution of folic acid fortification [24, 72]. The findings have stressed the need for more surveillance efforts, particularly in low-income countries [69]. In the current review, a relatively high-pooled birth prevalence of neural tube defects was detected in Algeria, Ethiopia, Eritrea, and Nigeria. Of all, the highest and lowest rates were detected in Algeria (75) and Tunisia (2), respectively. The magnitude of the defect among African countries showed geographic variations as other previous reviews have shown in various regions of the world [24, 69–75]. Thus, the variation detected across studies in estimating the pooled prevalence of neural tube defects was due to differences in study countries, period prevalence, study design, and birth outcome. The variation of estimates across countries may be also due to the difference in the folic acid supplementation/fortification, prenatal care/antenatal screening, and countries’ health policy.

The increment of prevalence over time may be due to a change in detection methods, an increment of the practices in documenting and reporting cases, an increase of the demands for fetal pathological examinations over these years, or a real increase in disease. Besides, it may be due to an increment of practice changes that could lead to increased detections, for instance, nowadays more children are born in hospitals and more women are became tested/screened.

Taking folic acid during early pregnancy had a non-significant association with the incidence of neural tube defects. However, this finding is not supported by different previous literature [24, 25, 29, 32]. Although folic acid has been revealed to decrease the risk of neural tube defects in previous studies [36, 37, 39, 76], the potential of folic acid to decrease the occurrence of the defect has not been yet examined in most African countries and preventable neural tube defects continue to occur [25]. Furthermore, the utilization is affected by the persistence of socioeconomic and educational issues in the consumption of folic acid, ethnic disparities, and the existence of age-based variation of supplement use [25]. Despite there are folate supplements, there is a low utilization, it is difficult to attain the recommended daily intake of folate for different reasons (relatively poor availability of folate in natural foods, easy destruction during cooking, for instance) [77]. May be the lack of significance is due to the inclusion of a small number of studies (and may be these are low folic acid utilized countries, non-mandatory folic acid users) in the analyses.

### Strength and limitations of the review

The present systematic review and meta-analysis gave cumulative and up-to-date evidence on neural tube defects and associated risk factors in Africa. The review finding is estimated from the pooled estimate of forty-three studies in Africa and it provides valuable information to the policymakers, and this should be the ultimate contribution of this review to the field.

The findings of the current review should be interpreted based on some limitations. The estimate did not consider the terminated pregnancies of the defect and this may reduce the pooled prevalence estimates. Moreover, the presence of significant variation across countries may underestimate the overall burden of neural tube defects in Africa. Underestimation of the burden of neural tube defects should be considered due to the missing of many stillbirths and home births that are delivered in the community setting. Furthermore, the variability of the sample size in the included studies might influence the pooled birth prevalence estimates. The risk factors are harder to assess given the limitations on that data. All studies in this review were institution-based studies. Although moderate publication bias was detected in prevalence estimates, we adjusted the bias using the trim and fill analysis.

## Conclusions

The pooled birth prevalence of neural tube defects in Africa was found high. A high-pooled prevalence of neural tube defects was detected in Algeria, Ethiopia, Eritrea, and Nigeria. The risk factors evaluated were not found significant.

We would like to inform policymakers that the pooled birth prevalence estimates are may be underestimated due to different mentioned factors and the pooled estimate should not impact policy decisions on prevention efforts negatively in Africa where policymakers may feel that this is not a big problem to prioritize the prevention funds. Strong prevention and control measures should be the priority. Moreover, limited available data on neural tube defects inform the need for additional primary, wide scope research that would improve the true burden of the defects and facilitate preventive policies on preventive factors in Africa.

## Supplementary Information

**Additional file 1: Supplementary file 1**. PRISMA reporting checklist

**Additional file 2: Supplementary file 2**. PubMed Searching methods

**Additional file 3: Supplementary file 3**. JBI critical appraisal checklists for all designs

**Additional file 4: Supplementary file 4**. Additional Table and Figures

## Data Availability

The data sets used and/or analyzed during the current systematic review and meta-analysis are included in the review and available from the corresponding author on reasonable request.

## Abbreviations

CI: Confidence Interval
DR: Democratic Republic
I^2^: I-Squared
JBI: Joanna Briggs Institute
MeSH: Medical Subject Heading
OR: Odds Ratio
PROSPERO: Prospective Register of a Systematic Review
PRISMA: Preferred Reporting Items for Systematic Reviews and Meta-Analysis
