# Supplementary material for: Birth prevalence of neural tube defects and associated risk factors in Africa: a systematic review and meta-analysis
Source: BMC Pediatr. 2021 Apr 21;21:190. doi: 10.1186/s12887-021-02653-9 (PMC8058994; doi:10.1186/s12887-021-02653-9)
Supplement: Supplementary file 2 — Additional file 2: Supplementary file 2. PubMed Searching methods [file 12887_2021_2653_MOESM2_ESM.docx]

**PubMed Searching Metheds**

| **S.no.** | **Searching terms** | **Number of articles** |
| --- | --- | --- |
| 1. | ("neural tube defects" OR "neural tube defects" [MeSH Terms]) AND (newborns OR neonates) AND (Africa) | **145** |
| 2. | ("neural tube defects") AND (newborns) AND (in Africa) | 137 |
| 3. | ("neural tube defects" OR "neural tube defects" [MeSH Terms]) AND (newborns OR neonates OR “live births” OR “stillbirths”) AND (Africa) | 153 |
| 4. | ("neural tube defects") AND (newborns) AND (Africa) | 63 |
