# Supplementary material for: Birth prevalence of neural tube defects and associated risk factors in Africa: a systematic review and meta-analysis
Source: BMC Pediatr. 2021 Apr 21;21:190. doi: 10.1186/s12887-021-02653-9 (PMC8058994; doi:10.1186/s12887-021-02653-9)
Supplement: Supplementary file 4 — Additional file 4: Supplementary file 4. Additional Table and Figures [file 12887_2021_2653_MOESM4_ESM.docx]

**S Table:** The burden of neural tube defects among African countries, 2020

| S. No. | Country | Prevalence of neural tube defects per 10, 000 births (95 % CI) |
| --- | --- | --- |
| 1. | Ethiopia | 61.43 (46.70, 76.16)⃰ |
| 2. | Tunisia | 2.00 (1.87, 2.13) |
| 3. | Nigeria | 32.77 (21.94, 43.59)⃰ |
| 4. | Algeria | 75.00 (64.98, 85.02) |
| 5. | Eritrea | 39.00 (32.88, 45.12) |
| 6. | DR of Congo | 10.00 (3.41, 16.60) |
| 7. | Sudan | 30.69 (24.02, 37.36) |
| 8. | Libya | 8.00 (3.61, 12.39) |
| 9. | Egypt | 16.00 (4.92, 27.08) |
| 10. | Cameron | 19.00 (15.28, 22.72) |
| 11. | South Africa | 10.45 (7.72, 13.19)⃰ |
| 12. | Ghana | 14.22 (10.33, 18.12) |
| 13. | Tanzania | 30.00 (24.19, 35.81) |
| 14. | Kenya | 3.00 (-1.37, 7.37) |
| 15. | Malawi | 6.00 (3.00, 9.00) |
| **Total** | **D+L pooled** | **21.42 (19.29, 23.56)⃰** |

⃰ : The difference within countries was statistically significant.

**Influence analysis**


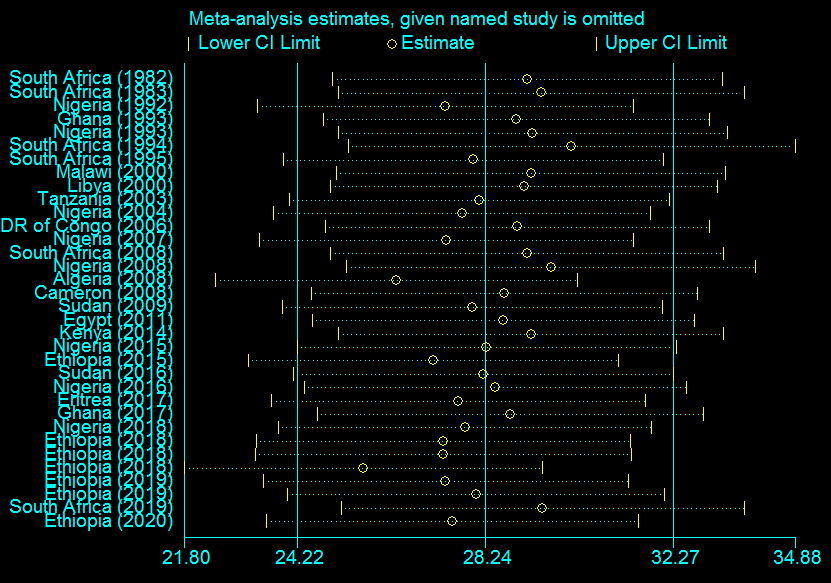


**S Figure 1:** Influence analysis after removing two studies (Tunisia 2014, 2015), 2020

**The estimates of high quality studies**

**S Figure 2:** The estimates of high quality studies (results after removing low quality studies) in Africa, 2020.

**Trim and fill analysis**


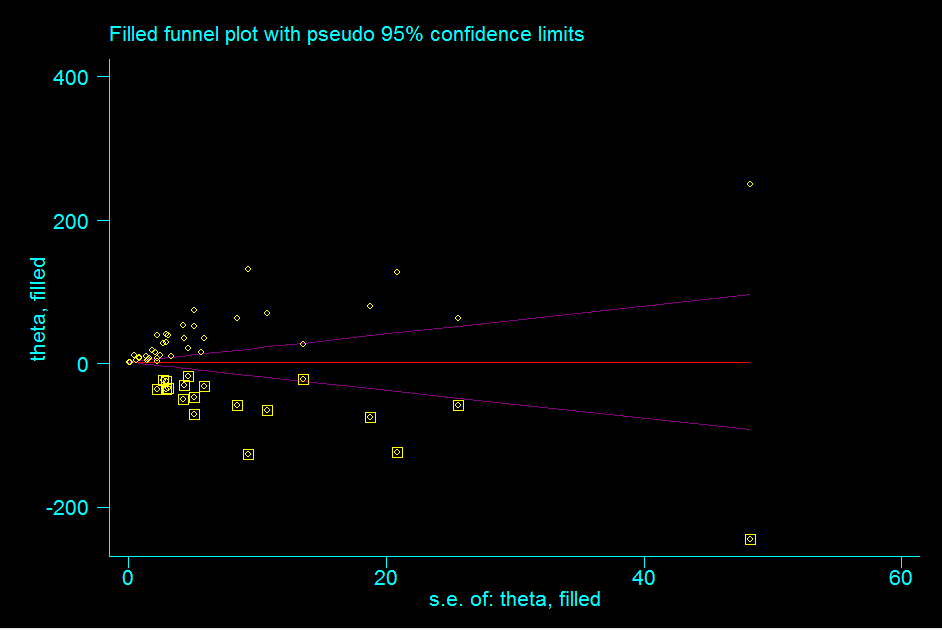


**S Figure 3:** Trim and fill analysis to minimize the publication bias among studies, 2020

**Taking folic acid**


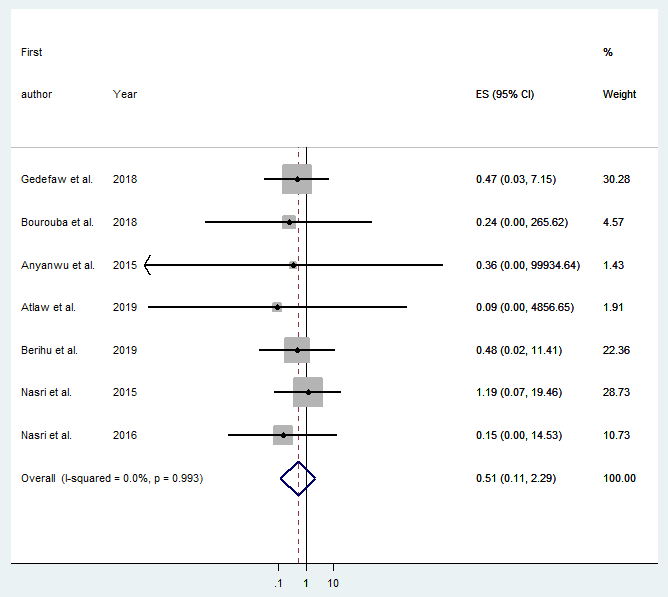


**S Figure 4:** Forest plot showing the pooled measure of association between neural tube defects and folic acid supplements, 2020

**Galbraith plot**


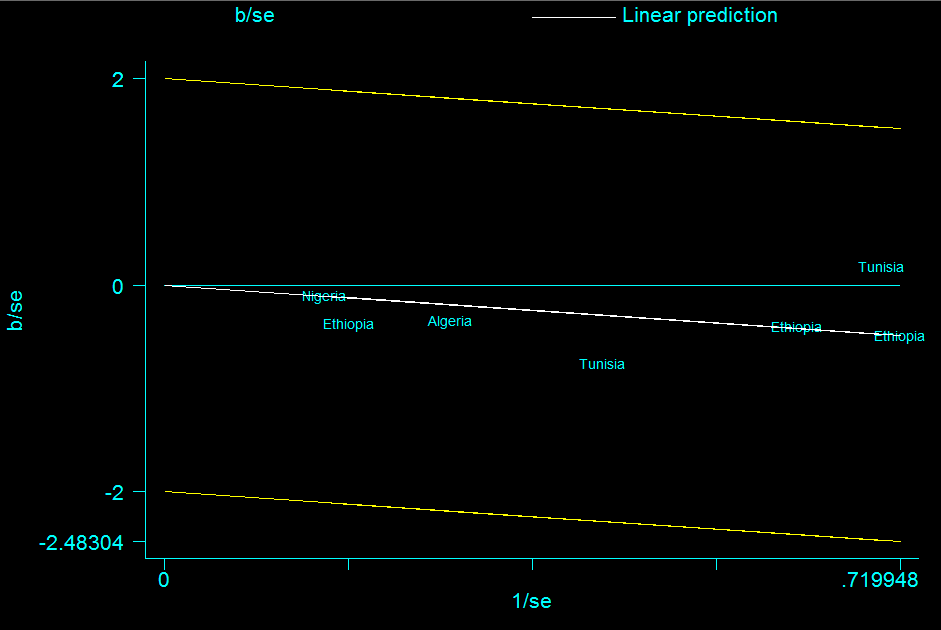


**S Figure 5:** Galbraith plot showing the variability of individual measure of association between neural tube defects and folic acid supplements, 2020

**Consanguineous marriage**


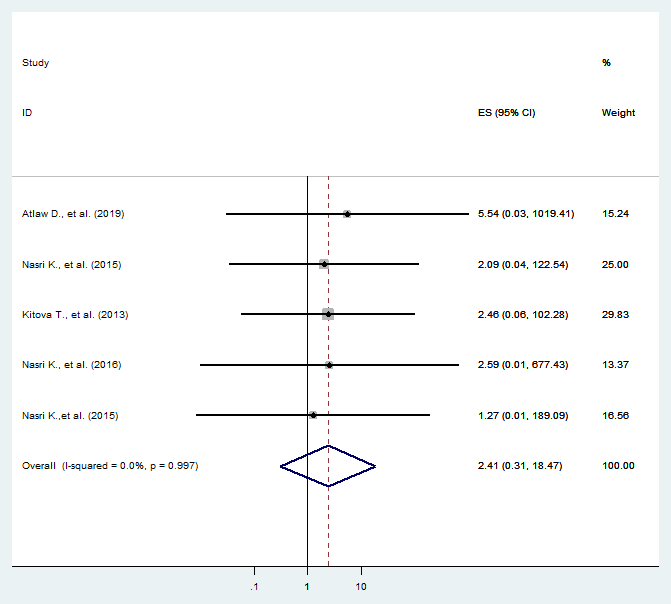


**S Figure 6:** Forest plot showing the association between neural tube defects and consanguineous marriage, 2020

**Male newborn**


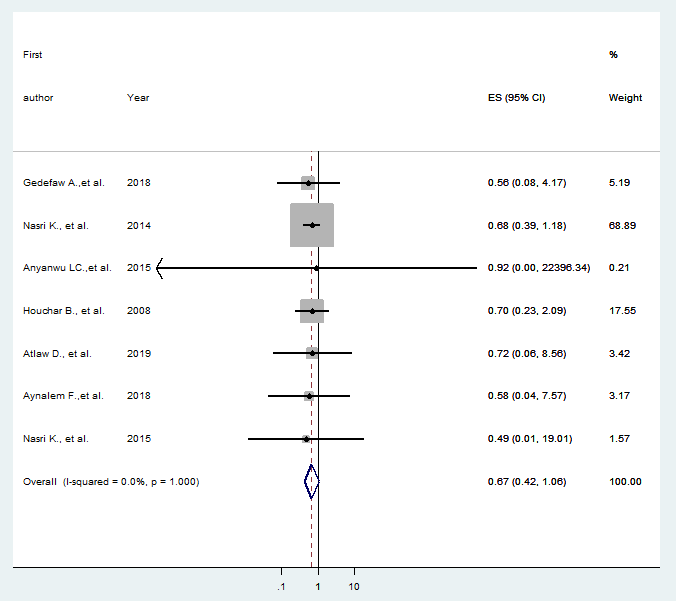


**S Figure 7:** Forest plot showing the association between neural tube defects and male newborn, 2020

**Substance use during pregnancy**


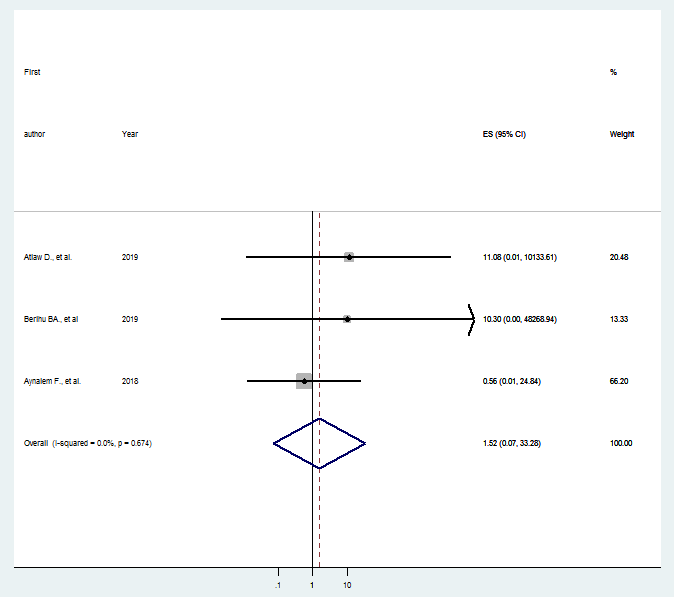


**S Figure 8:** Forest plot showing the association between neural tube defects and substance use during pregnancy, 2020
